# The Effect of Education and Implementation of Evidence-Based Nursing Guidelines on Infants’ Weight Gaining in NICU

**DOI:** 10.5539/gjhs.v7n2p148

**Published:** 2014-10-08

**Authors:** Zahra Salehi, Jamileh Mokhtari Nouri, Seyyed Mohammad Khademolhoseyni, Abbas Ebadi

**Affiliations:** 1 Behavioral Sciences Research Center (BSRC), Nursing Faculty, Baqiyatallah University of Medical Sciences, Tehran, Iran; 2 Nursing Faculty, Baqiyatallah (AJ) University of Medical Sciences, Tehran, Iran

**Keywords:** NICU, evidence-based guidelines, weight gaining

## Abstract

**Background:**

Educating evidence-based guidelines influences increased quality of nursing cares effectively. Infant’s weight gaining is one of the most important indicators for measuring quality of nursing care in NICU. The research is conducted with the aim of surveying the effect of education and implementation of educating evidence-based guidelines on infants’ weight gaining in NICU.

**Methods:**

This two-group clinical trial study was conducted in 2013 on one hundred infants in Baqiyatallah (AJ) hospital of Tehran. It was performed by using non-probable and convenient sampling. Data collection tools included; infants’ demographic questionnaire and a researcher-made checklist to record infants’ weight by using a weighing scale. Infants’ weight was recorded before intervention and two months after implementation of the guidelines, then data were analyzed by using SPSS19 statistical software.

**Findings:**

Mean weight of the infants in the control group on admission and on discharge was respectively; 1771(41.71) and 1712(42.68), and mean weight of the infants in intervention group on admission and on discharge was respectively; 1697(37.63) and 1793(40.71). After two months, infants’ weight gaining in intervention group was more than control group and it was statistically significant (P = 0.001).

**Conclusion:**

Results of the present study showed that implementation of evidence-based instruction an effective and economical method regarding infants’ weight gaining. Therefore it is recommended to the authorities and managers of the hospitals and educational centers of the healthcare services to put education and implementation of educating evidence-based instruction the priority of their work plans.

## 1. Introduction

Low birth weight causes infants’ death or long-term hospitalization in NICU (Field et al., 2011).

Premature and low birth infants are those that are born with weight less than 2500 grams. According to the statistics, death in these infants is forty times higher than infants weighing 2500 grams or higher than that (Mamun et al., 2011). Weight is the most important criterion among other evaluation criteria of the infants’ health. One of the common problems of the infants is being with low birth weight (Kulkarni et al., 2010).

Due to slow growth of a premature infant, weight increase is always considered as an important point and a difficult challenge, from the other side, lack of appropriate weight gaining is one of the important reasons of infant’s long-term hospitalization in NICU, increased costs, increased hospital infections, long-term complications of hospitalization and (Diego et al., 2014a).

Deborah (2012) named weight increase as one of the important aspects of managing premature infants in NICU and stated that weight increase is an important point; she believed that infant’s low weight leads to increased time of hospitalization (Steward, 2012).

Also many studies have shown that; improvement of growth and increase of weight are two important factors in decreasing death and early discharge of the infants from NICU (Bache et al., 2014).

Since performing intensive cares at premature and vulnerable infants’ bedside needs experienced and specialized people regarding infants’ care (Valizadeh et al., 2013); clinical team, especially nurses due to their important and vital role in increasing quality of the infants’ care should update themselves with the last evolutions regarding clinical cares and also they should update their clinical information (Considine & McGillivray, 2010). From the other side, promotion of care methods in these infants can cause decrease in their care costs, in addition to decrease of problems and complications in infancy, childhood and adulthood period. Because of this matter, evidence-based nursing discussion has been considered in hospital and educational centers in the recent years (Melnyk & Fineout-Overholt, 2011).

Educating evidence-based guidelines that are provided according to the newest researches have an important role in providing solutions and standardizing methods and they are counted as helpful tools and guides for the nurses of intensive units (Melnyk et al., 2014).

Evidence-based nursing performances are golden standards for providing nursing care. These performances are according to the evidences of the best researches, clinical experiences, expertise opinions, society standards, valid research evidences and patients’ approaches and values. Evidence-based clinical performance helps nursing sensitivity and efficiency and high quality of the health resultant to the high extent (Hong, 2010).

Massage therapy is one the evidence-based nursing performances for increasing premature infants’ weight. Massage therapy has been attracted by many supporters in intensive care units. In this regard, results of the study of Fallah et al. (2013) (Fallah et al., 2013) and Digo et al. (2014) (Diego et al., 2014b) showed that; massage therapy is an effective, economic and safe method for increasing premature infants’ weight.

Nurses should be aware of the way of using results achieved from the other studies for better provision of the cares and increasing their quality (Cullen & Adams, 2012). Correct usage of the evidences and findings of other studies lead to quality promotion and makes the nurses responsible for their performance. But the conducted articles and studies in this regard show that most of the medical personnel are not familiar with evidence-based guidelines, they are not aware of the effect of performing these guidelines on nursing care quality and they do not use results achieved from the conducted studies a lot (Badr et al., 2011).

For maintaining and developing these standards, it is necessary for the personnel to develop and organize their knowledge; this issue needs permanent in-service education and updated information. Petty Jollea in 2010 stated that in-service education of the nurses working in NICU leads to increased nursing cares quality (Petty, 2011).

Evidence-based clinical guidelines are a valuable source for clinical work especially in intensive care units that nurses have an important role in managing clinical work. Since in our country, medical personnel are not familiar with evidence-based guidelines, they do not use results achieved from such studies. Therefore, the aim of this study was determining the effect of education and implementation of educating evidence-based guidelines on infants’ weight gaining in NICU.

## 2. Materials and Methods

This is a before and after two-group clinical trial study. This study is conducted in NICU Baqiatallah General Hospital of Tehran in 2013. 100 samples were estimated by using Morgan table, fifty infants were in before intervention group (control group) and fifty infants were in after intervention group (experimental group). Before beginning of the study, the permission to conduct research was taken from researches committee of Baqiatallah (AJ) Medical Sciences University. Also samples participating in the study were informed about the aims and the method of the study and that their participation in the study is voluntary and they can stop their participation at any time. Non-probable and convenient sampling method were used for the infants, in this way that infants with inclusion criteria such as infants less than 37 weeks of gestation without any long-term complications of associated diseases, infants without any anomalies or syndrome and weight less than 2500 grams were selected by referring to NICU. Infants that had a crisis during treatment process such as being coded were excluded from the study.

Data collection tools included: infants’ demographic questionnaire such as; age, gender, hospitalization duration…and the infants’ weight data sheet by the weighing scale with Seca mark. The scale was calibrated at regular intervals. The weighing scale wase made by Seca (Germany). At first, all babies were weighted by an infant digital weighing scale with a sensitivity of 10 g without diapers; by a special person through using fixed digital scale at a determined time.

After collecting data in the control group, twenty evidence-based guidelines regarding infants and the method of using and recording them according to the nursing process procedures (diagnosis, planning, implantation and evaluation) were taught to the personnel by the project supervisor in two four-hour educational workshops and the importance of performing these guidelines was emphasized.

Topics of the implemented guidelines included; impaired gas exchange, ineffective thermoregulation, infection risk, altered nutrition less than body’s need, fluid valium deficient, high risk for impaired skin integrity, decubitus ulcer, altered family processes, pain, sensory overload, delayed growth and development, grieving, risk of impaired attach, risk of sudden infant’s death syndrome, ineffective infant’s feeding pattern, parental anxiety, deficient knowledge parental, aspiration risk, neonatal jaundice, ineffective breastfeeding.

Education content also included; explanation and definition of educating evidence-based guidelines, explanation and definition of nursing process procedures and the way of its chart, explanation of different kinds of nursing process procedures recording sheets and nursing care sheet and discharge sheet. For resolving the possible problems in recoding sheets, one session was held for resolving problems in the next two weeks. The researcher controlled recordings through his permanent presence; the done corrections were explained to the nurses by the help of the research team. Two months after education and implementation of guidelines, infants’ weight was recorded for the second time and before and after intervention results were compared. Data were assessed by SPSS 19 software and by using average and standard deviation, independent t-tests and chi-square.

## 3. Results

Comparison of demographic variables in the two groups in Table 1 shows that the two groups are in consistency in this regard (Table 1).

**Table 1:** Frequency distribution of the infants’ demographic features in the two groups before and after intervention

Frequency	Criteria	Control group Number (percent)	Intervention group Number (percent)	Experimental	Significant level
Gender	Female	27(54)	16(32)	Chi-square	x^2^=4.937 P= 0.26
	Male	23(46)	34(68)		
Age	0-10 days	3(6)	0(0)	Chi-square	4.334=x^2^ 0.115=p
	11-20 days	35(70)	32(64)		
	21-30 days	12(24)	18(36)		

The average weight of the infants in control group on admission and on discharge was respectively: 1771 (419.718) and 1712 (422.686) and the average weight of the infants in experimental group on admission and on discharge was respectively: 1697 (379.639) and 1793 (403.719) (Table 2).

**Table 2:** Comparison of the average of infants’ weight in the two groups

Weight (gram) group	Measurement time		The mean difference	Independent t-test

	On admission to NICU	On discharge from NICU		

	Average (standard deviation)	Average (standard deviation)	Average (standard deviation)	
Control group	1771(419.7)	1712(422.6)	-59(95.1)	t =-8.2 df= 98
Intervention group	1697(379.6)	1793(403.7)	+96(92.8)	P=0.001

## 4. Discussion

Results of the present study showed that; education and implementation of evidence-based guidelines lead to significant weight increase in intervention group rather than control group.

Ineffective infant’s feeding pattern was one of the nursing diagnoses in the present study. This guidelines will be used in the case of infant’s inability of consuming food stuffs because of disease or prematurity. Gavage feeding is one of the most common nursing measures in this regard.

Results of the study of Street et al. (2006) showed that performing nutrition guidelines in premature infants leads to their increased weight and decreased hospital stay.

Growth and development delay or ineffective infant’s feeding pattern were among other nursing diagnoses in the present study. In this regard, massaging infant’s body is one of the important nursing measures for increasing premature infant’s weight, which is highly taken into consideration by the researchers.

There are many studies regarding the effect of massage on premature infants’ growth; it can be used alone or with different types of vegetable oils. Sankaranarayanan et al. (2005) and Javadifar et al. (2009) (Javadifar et al., 2009) stated that in this respect, massaging body with coconut oil is an effective and certain method for increasing premature infants’ weight. Also Basiri et al. (2006) showed that intervention of touching can be an economic method for facilitating premature infants’ growth (Basiri et al., 2006). Also Saeedi et al. (2009) (Saeidi et al., 2009) in their study considered massaging premature infants’ body with oils containing triglycerides as a way for infants’ rapid weight gaining.

Masaro et al. (2009) in a clinical trial study showed that massaging premature infants’ body is an economic method for increasing their weight and decreasing their hospitalization period (Massaro et al., 2009).

Results of another study which was done by Fallah et al. (2013) on 54 premature infants hospitalized in NICU showed that massage with sunflower oil is an effective and safe method for increasing premature infants’ weight (Fallah et al., 2013).

Also results of the study of Digo et al. (2014a) showed that massaging premature infants’ body is an effective and safe method for increasing their weight (Diego et al., 2014b). Results achieved from these studies were all in consistent with the present study. These results indicated that evidence-based nursing care is a useful method for increasing premature infants’ weight.

Premature and low birth weight infants are exposed to high risk of feeding problems due to prolonged hospitalization and limited opportunities for sucking. Altered nutrition; less than body requirement was one of the performed nursing diagnoses in our study. Among effective nursing measures in this regard, it can be pointed out to non-nutritive food for stimulating sucking power in premature infants that can’t beefed orally, this method makes a chance for them to learn sucking for successful feeding.

BehnamVashani et al. (2013) by assessing the effect of non-nutritive sucking on premature infants’ weight showed that non-nutritive sucking did not influence weight increase remarkably; even it may cause energy expenditure and weight loss. They believed that long time use of non-nutritive sucking may lead to weight increase (Voshani Hb, 2013) results achieved from the present study were in contrast to our findings.

## 5. Conclusion

Implementing and educating evidence-based guidelines were effective in increasing infants’ weight gaining. Therefore, it is recommended to the authorities and managers of the hospitals and educational centers of health services to put education and implementation of evidence-based guidelines in the priority of their work plans.
